# Post-traumatic Trigeminal Neuropathy Associated With Endodontic Therapy: A Systematic Review

**DOI:** 10.7759/cureus.32675

**Published:** 2022-12-18

**Authors:** May W Al-Khudhairy, Ghada Albisher, Alhanouf Alarfaj, Saffanah Alabbadi, Najibah Almohaishi, Walaa Alqudaihi

**Affiliations:** 1 Oral Biology, Riyadh Elm University, Riyadh, SAU; 2 College of Dentistry, Riyadh Elm University, Riyadh, SAU

**Keywords:** persistent idiopathic facial pain (pifp), phantom tooth pain, atypical facial pain, neuropathic orofacial pain, atypical odontalgia (ao), persistent dentoalveolar pain (pdap), post-traumatic trigeminal neuropathy (pttn)

## Abstract

A painful or non-painful trigeminal nerve lesion brought on by trauma that exhibits symptoms and/or clinical evidence of trigeminal nerve dysfunction is known as painful post-traumatic trigeminal neuropathy (PTTN). In relation to this, the term post-traumatic persistent dentoalveolar pain (PDAP) is an idiopathic condition of chronic neuropathic origin that manifests as a diagnostic challenge for dental practitioners. Neuropathic pain is defined by the International Association for the Study of Pain (IASP) as "pain initiated or caused by a primary lesion or dysfunction in the nervous system." PDAP is located primarily in the teeth and jaws. This study systematically reviews how likely it is to get painful PTTN if the patient received endodontic therapy and the duration between doing root canal therapy (RCT) and getting PTTN. A systematic review was carried out using key search terms from PubMed, Web of Science, and the Cochrane Central Register of Controlled Trials (CENTRAL) with English as the only permitted language. There were strict inclusion requirements. The 10 articles that were included showed a prevalence of an endodontic procedure anywhere from three to 48 months following post-endodontic treatment, and it mainly affects females in their mid-40s with no variation regarding the areas, whether it is in the maxilla or mandible. The lack of information about the association between RCT and PTTN led practitioners to make wrong diagnoses, which made the patient unwilling to seek further help. So, in this review, we identified some visible characteristics that can help in that process.

## Introduction and background

Persistent dentoalveolar pain (PDAP) is an idiopathic condition of chronic neuropathic origin that manifests as a diagnostic challenge for dental practitioners [1]. Several endeavors are needed to identify the origin of PDAP [2]. Three different root cause origins are discovered, which are psychogenic, vascular, and neuropathic [3]. Neuropathic origin is declared to be the primary due to the phenomena of deafferentation [3]. The loss of the spinothalamic tract, which transmits somatosensory information regarding pain, itch, and rough touch, causes deafferentation [4]. Even after the wounds have fully healed, deafferentation of nerve fibers, which usually occurs due to a severe injury, causes ongoing discomfort, paresthesia, and dysesthesia [3]. PDAP is located primarily in the teeth and jaws [5]. PDAP can originate after multiple invasive dental treatments, such as tooth extraction, bone grafting, dental implantation, apicoectomy, and endodontic treatment [5,6]. It was initially discovered by McElin and Horton in 1947, and from that point forward, there have been numerous clinical cases in the literature, particularly related to root canal therapy (RCT) [3]. PDAP has a variety of manifestations. The initial symptom is intraoral unilateral pain but, with time, the pain extends bilaterally in up to one-third of the patients, which could be deep or superficial, localized in any tooth or mucosa [7]. However, as time passes, the pain may spread and become generalized [7]. Pain may vary but is usually experienced as aching, burning, or throbbing [7]. The intensity of pain ranges from mild to severe [7]. Patients may find intervals of pain relief during the frequently ongoing but intermittently intense pain [7]. The beginning of pain that frequently follows dental treatments, such as tooth extraction, endodontic therapy, and sinus surgery, is triggered by a temperature change [7]. Moreover, the pain may occur without preceding events or in the absence of dental causes [7].

The duration of persistent idiopathic facial pain, which includes atypical odontalgia (AO), could last for more than two hours up to three months, despite the absence of neurological defects [7]. PDAP most commonly affects the upper jaw molars and premolars [7]. Females have been seen to predominate in 65-85% of PDAP cases [8]. The average age range is 15-80 years for female patients and 23-69 years for male patients [9]. The misdiagnosis of this condition is mainly caused by the lack of clinical and radiographic corroboration [1]. Prior to beginning any treatment, an accurate diagnosis must be performed [1]. Negligence in achieving the correct treatment has a negative impact on the quality of the patient’s life [2]. Therefore, the patient’s struggle is due to insufficient knowledge of the dental practitioner and incorrect treatment proposals [1]. Brooke and Merskey supported this statement by describing the role of emotional alterations as a contributing factor to AO [3]. This condition was named earlier in 1974 as "idiopathic periodontalgia" [1]. In 1978, Marbach termed this condition "Phantom Tooth Pain." The term "atypical odontalgia" first appeared in 1978 and, since then, it has been more commonly used terminology by the International Headache Society (HIS) and the International Association for the Study of Pain (IASP). The best terms to use would be AO and PDAP because they do not imply causative hypotheses [1]. The HIS has recently proposed the name painful post-traumatic trigeminal neuropathy (PTTN) to describe neuropathic pain with a traumatic etiology that affects the trigeminal nerve. This phrase describes a painful neuropathy that affects the face, mouth, and other areas of the head [10]. The goal of our study is to systematically review the possibility of developing PTTN if the patient received endodontic therapy and the time interval between endodontic therapy and PTTN. 

## Review

Level 1 and level 2 evidence with a low-to-moderate risk of bias were the criteria for the complete peer-reviewed search process, which was available in an online database. All studies in the English language that link human endodontic patients and painful PTTN were included. This includes cohort studies, cross-sectional studies, prospective studies, retrospective studies, and randomized controlled trials. High-quality research on this subject was lacking. The Institutional Review Board of Riyadh Elm University has examined the review (registration number FRP/2020/278/293 (REU)).

Each of the following internet databases was searched to find potential studies to include in the systematic review: PubMed, Web of Science, and the Cochrane Central Register of Controlled Trials (CENTRAL). Using the terms painful PTTN, PDAP, AO, neuropathic orofacial pain, atypical facial pain, phantom tooth pain, and persistent idiopathic facial pain, we conducted an online literature review search for trigeminal nerve injury. The search was limited to articles in the English language. We looked over the words in the abstract and title fields. References from works that matched the requirements for inclusion as well as those from excellent reviews of the subject were examined.

Studies were included if they included information on patients who had received a diagnosis of PTTN in conjunction with RCT, provided information on the diagnostic procedures and standards utilized to identify PTTN following RCT, PTTN or unpleasant sensation in regions supplied by the trigeminal nerve had been diagnosed in a sample, were published between 2009 and 2020, and were human subject studies.

Studies were disregarded if they dealt with animal subjects in a laboratory setting or research that is unrelated to the study's topic, were conference and book abstracts and article reviews, were written in languages other than English, or were published prior to 2009.

Outcome measures

Table 1 represents the primary outcome of this investigation. This study systematically reviews how likely it is to get painful PTTN if the patient received endodontic therapy, the time between the endodontic procedure and diagnosis of AO, and educates the public and health practitioners.

**Table 1:** PICO components. RCT: root canal therapy; PTTN: post-traumatic trigeminal neuropathy; PICO: population, incidence, comparison, and outcome.

Variables	Questions
Population	Patients with RCT
Incidence	Patients with PTTN
Comparison	Compare RCT patients without PTTN/PTTN patients
Outcome	Prevalence of PTTN after RCT

Data collection and analysis

**Figure 1:**
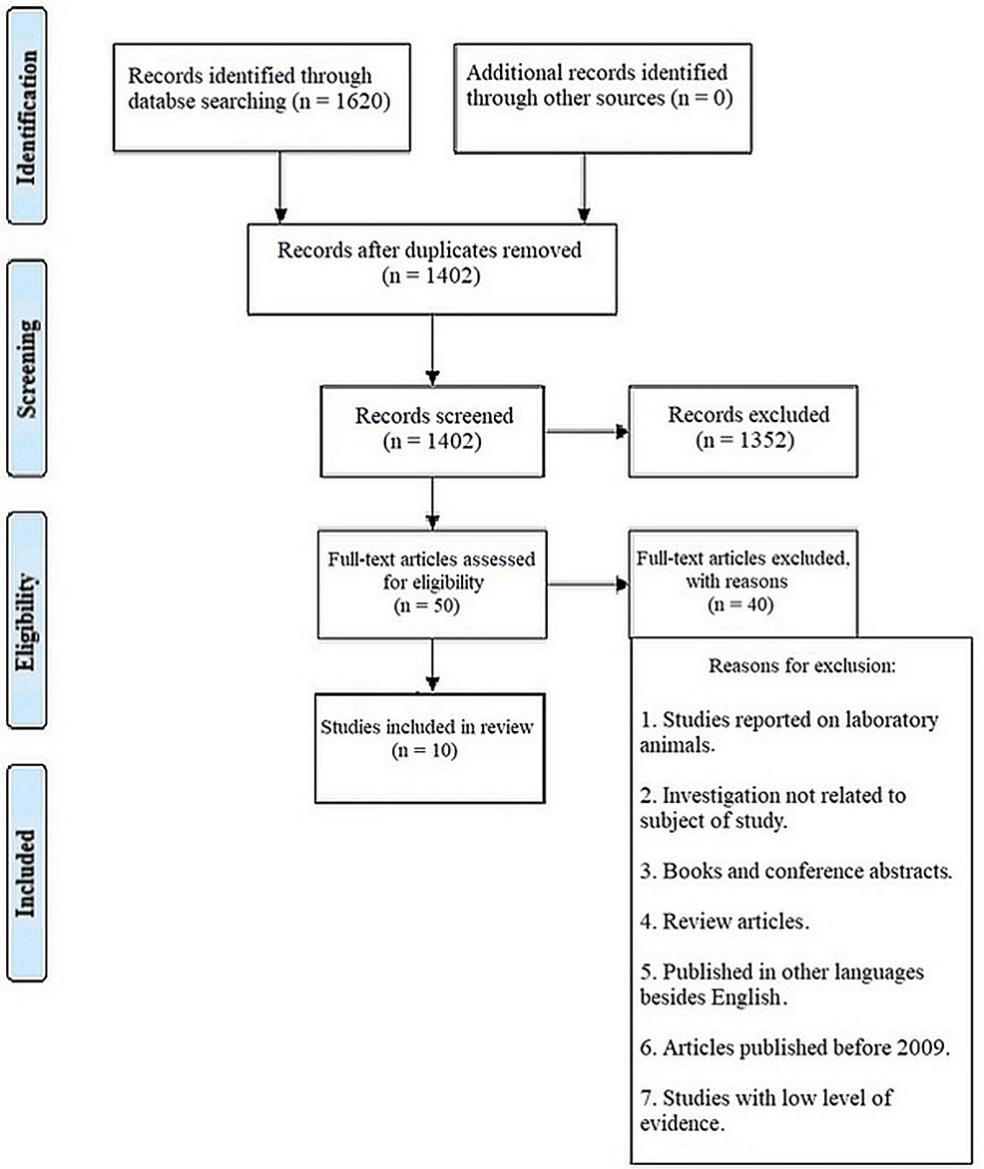
The PRISMA flow diagram details the search and selection process applied during our systematic search. PRISMA: Preferred Reporting Items for Systematic Reviews and Meta-Analyses.

Utilizing an electronic database, the references of the papers were collected as represented in Figure 1. To determine whether the articles fulfilled the inclusion criteria, five independent reviews were conducted. If consensus could not be achieved, the article's eligibility was decided after consulting a third reviewer. The five primary reviewers then read the entirety of each paper that appeared to fit the inclusion criteria after screening the papers. All studies that met the inclusion criteria had their validity assessed and their data extracted.

Data were extracted and independently validated by the fourth and fifth reviewers from each manuscript that satisfied the inclusion criteria as decided by the first, second, and third reviewers. The database was updated with the first and second reviewers, and the third, fourth, and fifth reviewers verified the correctness of the document. The data for each included article is recorded in Table 2. There were 10 articles altogether. Data analysis was done, and the results reflected all citations located using electronic search techniques.

**Table 2:** Data of included articles PTTN: post-traumatic trigeminal neuropathy; NSRCT: nonsurgical root canal therapy; RCT: root canal therapy; CBCT: cone beam computed tomography; OHRQoL: oral health-related quality of life; IA: inferior alveolar; PDAP: persistent dentoalveolar pain; VAS: visual analog scale; AO: atypical odontalgia; PEARL: peer experience and reflective learning; CTN: classical trigeminal neuralgia; PPTTN: peripheral painful traumatic trigeminal neuropathy; TMD: temporomandibular disorder.

Title	Author/year	Patients/country	Study design	Sample size Male/female	Age	Time between diagnosis of PTTN and endo RCT	How PTTN diagnosed	Results
1,331 patients with PTTN who were seen in two tertiary referral centers in two different countries were evaluated for signs and symptoms, quality of life, and psychosocial data	Van der Cruyssen et al./2020 [11]	1,331/Belgium and England	Retrospective/clinical trial	405 Males (30%) and 926 females (70%)	Mean age 46 years	112 days	Neurosensory testing	Third molar was the most common culprit for 519 (48%) cases of trigeminal nerve damage, followed by dental implants in 146 (13%) cases, extraction of non-third molar in 136 (13%) cases, local anesthesia in 125 (12%), endodontic treatment in 86 (8%), and trauma in 74 (7%), with a mean P value of 86. Arch: (Not mentioned) Gender: Females more. Age: Mid-40s
Clinical examination of patients with neuropathic tooth pain following endodontic treatment	Oshima et al./2009 [12]	271/Japan	Clinical research/retrospective study	16 Endodontic follow-up patients that included 13 Females and three males	Mean age 46.8 years	One to two months	Clinical examination Used microscopic If needed used CT Clinical interview Diagnostic LA at the site of pain	The majority of the patients (14) reported maxillary discomfort, while only two (12.5%) had mandibular pain. In 10 (62.5%) and five (31.3%) of the 16 patients, respectively, the onset of pain was correlated with retreatment or pulp extirpation. One patient made no explicit mention of how their discomfort first started. Arch: (Maxillary arch was mentioned to have more pain in clinical cases but not addressed clearly) Gender: Females were mentioned to fulfill the criteria. Age: Mid-40s.
A prospective study looked at the prevalence, risk factors, and clinical trajectory of chronic pain in teeth that had nonsurgical RCT and showed signs of periapical healing	Philpott et al./2018 [13]	198 Patients 264 teeth/London, UK	Prospective study/retrospective/clinical	Not specified	≤16 Years	Five to 14 months post-NSRCT	Clinical examination CBCT McGill Pain Questionnaire	20% of the sample size exhibited persistent pain. Predictive factor: Following RCT, female gender, a history of chronic pain, teeth that responded to a pulp test, teeth linked to preoperative pain, tooth cracks that required treatment before they occurred, and the diameter of preoperative radiolucency. Arch: not mentioned only said whether multiple teeth received RCT adjacent to each other or in the same or opposing arch. Age not mentioned
The frequency of ongoing discomfort following non-surgical RCT	Klasser et al./2011 [14]	250 Adults/ USA	Retrospective study	138 Females and 112 males	Common in middle age (mean age 50.6 years)	Four years	Clinical examination VAS, Self-administered Leeds Assessment of Neuropathic Symptoms and Signs	The prevalence of neuropathic pain is higher in middle-aged people without any preference for sex and is higher in the mandibular arch regardless of the number of canals that have been treated.
Findings from the PEARL Network on the prevalence of persistent pain three to five years after primary RCT and its effect on OHRQoL	Vena et al./2014 [15]	63/New York, USA	Retrospective study/clinical	24 odontogenic pain: 16 females 8 males 39 non-odontogenic pain: 18 females 21 males	Odontogenic pain mean age 52.5 years Non-odontogenic pain 50 years	≥6 months	Clinical examination Radiographs Tooth sensitivity assessment Standardized OHRQoL Questionnaire	Odontogenic pain: Maxilla 11 Mandible 13 Non-odontogenic pain: Maxilla 22 Mandible 17 Sex, age, and arch were not found to be associated with non-odontogenic pain The outcomes after RCT, 3.1% of patients had pain unrelated to odontogenic reasons.
PTTN	Peñarrocha et al./2012 [16]	63/Spain	Retrospective case study	11 Males and 52 females	Mean 45.4 (17-76) years	At least six months	Clinical examination Pain intensity level	Impacted lower third molar removal (34 patients, 54%), simple extraction (9 patients), endodontic treatment (1 patient), pain reported by four males and 32 females in their mid-40s, arch not mentioned, age not mentioned
Clinical characteristic and diagnosis of AO	Ram et al./2009 [17]	64/USA	Retrospective observational study	44 Females and 20 males	55.4 Years	Six months	Radiographic investigation Anesthetic testing	Endodontic patients have AO in 3%-6% High prevalence in women in 40 No. of patients with AO from Endo tx = <5 patients
Iatrogenic trigeminal post-traumatic neuropathy: a retrospective two-year cohort study	Klazen et al./2018 [9]	University Hospitals of Leuven In Belgium	Retrospective cohort study	8,845 patients 8,845 new patients 53 cases of iatrogenic trigeminal nerve injury female (n = 36, 68%) male (n = 17, 32%)	Average age was 42.9 years (range 15–80 years) for female patients and 40.4 years (range 23–69 years) for male patients (overall average age 42.1 years)	2013-2014	Screen record included demographic data	Patient related to endodontics: 6%. Arch: Not mentioned. Gender: Females more. Age: Mid-40s
Differential diagnoses for persistent pain after root canal treatment: a study in the National Dental Practice-based Research Network	Nixdorf et al./(2015) [18]	(Alabama/Mississippi, Florida/Georgia, Minnesota, Permanente Dental Associates in Oregon/Washington, and Scandinavia (Denmark and Sweden))	Prospective longitudinal cohort study	38 of the 354 eligible patients in the geographic area (11%) met the pain criteria female (84%) male not mentioned	19–70 years The mean age of patients with pain was 49 (standard deviation 13) years	Six months	Clinical evaluations include periapical and CBCT radiographs	Pain after six months of RCT: non-odontogenic pain: eight patients (seven from referred TMD pain and one from PDAP disorder), mixed: two patients (one PDAP, one TMD). Arch: Maxillary teeth compromised 53% of treated teeth, and 89% were posterior teeth. Gender: Only females mentioned. Age: 40s
Clinical features in 91 cases of PPTTN and the proposal of novel diagnostic criteria	Benoliel et al./(2012) [19]	University–Hadassah Jerusalem, Israel	Prospective cohort study	A total 145 *91 cases of PTTN *54 cases with CTN Gender (male:female) 34:57	PTTN (48.60 ± 15.2 years)	Minimum period three months	Radiographs Application of the cold test	PPTTN criteria can detect and characterise relevant cases clinically. The observed profile included continuous and paroxysmal stabbing and/or burning pain, unlike PPTTN. CTN patients had different quality, duration, and intensity (P < 0.05). 96% of PPTTN cases had trigeminal sensory abnormalities, mostly allodynia, hyper or hypoalgesia, and 1% had anaesthesia.

Study characteristics

Of the 10 articles that were chosen, two (20%) were retrospective clinical trials, four (40%) were retrospective studies, one (10%) was a retrospective observational study, one (10%) was a cohort study that was retrospective and prospective, one (10%) was a prospective cohort study, and one (10%) was a prospective longitudinal cohort study. Each research included anywhere from eight to 1,331 individuals. The mean age ranged from 40 to 55.4 years, and the gender distribution ranged from 31% male to 64% female. The median age of PTTN is 40 years. The prevalence of neuropathic pain in the maxillary arch was reported in just two (20%) of the 10 research, whereas the mandibular arch was mentioned in two (20%) of the 10 studies. The interval between the onset of PTTN and the start of RCT is 3-48 months.

Diagnostic tests for PTTN

Extraoral examination of the face, head, and neck, periapical radiographic examination, asymmetry examination, tender points, auscultation and palpation of the temporal-mandibular joints, and evaluation of mandibular movements were the clinical examination tests used in our study. During the intraoral examination, the patients' occlusion and any interferences with the root-filled teeth were evaluated. The existence or absence of a swelling, sinus tract, periodontal probing depths, pain to pressure or percussion on the tooth, and integrity of the restoration margin was among the clinical data noted. The most often reported diagnostic procedures (any signs or symptoms emanating from adjacent teeth were examined) were reported in eight (80%) of the 10 included papers. Of the 10 examined studies, only one study (10%) used clinical interviews to determine whether the pain "rarely interrupted sleep" and was "non-responsive to analgesics such as non-steroidal anti-inflammatory medications (NSAIDs)." To check for the presence of tissue hyperesthesia around the painful area, a diagnostic local anesthetic (LA) was used. Two (20%) of the 10 included papers addressed this test; hence, they were included. Cone beam computed tomography (CBCT) was used to assess the likelihood of differentiating between symptomatic apical periodontitis and "atypical odontalgia." One (10%) out of every 10 articles that were included provided an illustration of the Pain Catastrophizing Scale and the short-form McGill Pain Questionnaire (SF-MPQ). Visual analog scale (VAS), a numerical rating scale with anchors ranging from 0 (no pain) to 10 (pain as severe as it may be), and the Self-administered Leeds Assessment of Neuropathic Symptoms and Signs (S-LANSS) survey are used to determine who had chronic pain (longer than three months). Both were depicted in two (20%) of the 10 articles that were included: a standardized Oral Health-Related Quality of Life (OHRQoL) survey and an evaluation of tooth sensitivity (Oral Health Impact Profile-14 (OHIP-14)). The most common self-reported tool used by people to evaluate disease limitations associated with oral problems is the OHIP-14. It has great reliability and validity and is divided into seven sections: functional limitation, pain, psychological discomfort, physical, psychological, or social disability, and handicap. These assessments were in one (10%) of 10 included articles. The pain intensity level was mentioned in one (10%) of 10 included articles. Computed tomography (CT) for other diagnoses was mentioned in one (20%) of 10 included articles.

Discussion

The initial goal of this comprehensive study was to determine how often a patient develops PTTN after receiving endodontic treatment, secondly the time between the endodontic procedure and diagnosis of PTTN, and finally to educate the public and health practitioners on research methods for the identification of the study. Six of the 10 papers that were included utilized the phrase "atypical odontalgia" as their terminology. Although there are substantial differences in how these terms are employed in different studies, terminology like PTTN is routinely used to refer to all of the included studies.

In eight of 10 trials, several clinical examination tests were employed. The extra-intraoral examination was one of them. A clinical examination of the face, head, and neck was performed during the extraoral examination (asymmetry, tender points, auscultation, palpation of the temporomandibular joints, and assessment of mandibular movements). During the intraoral examination, the patient's occlusion and any interferences with the root-filled teeth were evaluated. The existence or absence of a swelling, sinus tract, periodontal probing depths, pain to pressure or percussion on the tooth, and integrity of the restoration margin were among the clinical data noted. Any symptoms or signals coming from nearby teeth were evaluated and taken into account [13]. A structured interview with questions about the patient's medical history, a review of the patient's medical records, and a methodical assessment of the patient's teeth and other oral structures were all included in the percussion, replacement status of the restoration, the need for replacement, the number of proximal contacts, the existence of fractures or cracks in the teeth, primary or secondary caries, and the presence and severity of periodontitis. The factors under investigation included (a) gender and age, (b) pain location, (c) pain duration, (d) pain severity, (e) triggering event, and (f) other clinical signs or symptoms during the initial examination. On every visit, the patient's pain levels were systematically recorded using a numeric rating scale (NRS). On the NRS, degrees of pain intensity ranged from 0 to 10, with 0 denoting perfect lack of pain and 10 denoting the worst possible suffering. Every patient's level of discomfort was noted in a chart both before and after treatment [12]. In every instance, extraoral panoramic X-rays were taken. In certain instances, CT scans of the maxilla, cranium, or both were done. In addition to the vitamin B complex, amitriptyline (50-75 mg/day) and/or carbamazepine (400-600 mg/day) was recommended for pain [16].

A routine physical examination of the cranial nerves, dental and periodontal tissues, the head and neck, and the masticatory apparatus (temporomandibular joints and masticatory/neck muscles) was recommended. The studies made an effort to pinpoint tactile trigger points or locations where pressing triggered excruciating pain that traveled beyond the point of stimulation. The following muscles were examined bilaterally: temporalis, masseter, medial pterygoid, sternocleidomastoid, trapezius, and the suboccipital (as one group). Muscle trigger points were included as part of the assessment of muscle involvement, not to be confused with the tactile trigger areas described for classical trigeminal neuralgia (CTN) and peripheral painful traumatic trigeminal neuropathy (PPTTN) [19]. Following standard procedures in each discipline, the clinician independently took a thorough history, conducted a clinical examination, and reviewed all of the patients' periapical radiographs. Clinical evidence confirming a non-odontogenic diagnosis included tenderness to palpation on the masseter, temporalis, and lateral pterygoid muscles as well as the temporalis tendons, recreating one aspect of the patient's complaints of ongoing pain. These patients met the referral criteria for the TMD diagnosis of myofascial pain. Allodynia, or a positive reaction to sensory tests, indicated the presence of nerve dysfunction and supported the diagnosis of PDAP [11], along with the usage of complex quantitative sensory tests (QST), demographic data, and neurosensory testing [18].

Anesthetic Test at the University of Southern California Orofacial Pain and Oral Medicine Clinic

To protect the sore area, cotton rollers and a cheek retractor are used. The sore spot is dried with a piece of 2 cm by 2 cm gauze. The patient's level of discomfort is measured using a 0-10 VAS. Benzocaine 20% gel is topically applied to the sore spot. The patient's pain is recorded on the VAS every three minutes. A LA infiltration or nerve block injection with 2% lidocaine gel is administered to the painful region if the pain alleviation is insufficient. Additionally, after three minutes, the pain level is recorded on the VAS. During a subsequent visit, the test is repeated [17]. The techniques for PTTN utilized in various investigations likewise varied greatly. We discovered that the diagnostic criteria put forth by Graff-Radford and Solberg in 1992 are the ones that best describe the condition (Table 3) [20].

**Table 3:** Diagnostic criteria for AO. AO: atypical odontalgia.

Idiopathic toothache (AO)
Pain in a tooth or tooth site.
Continuous or almost continuous pain.
Pain persisting more than four months.
No signs of local or referred pain.
Equivocal somatic nerve block.

A third molar was found to be the most frequent cause of trigeminal nerve damage in 519 (48%) females in their mid-40s according to a study by Van der Cruyssen et al. [11]. The above finding was followed by dental implants in 146 (13%) cases, third molar extraction in 136 (13%) cases, local anesthesia in 125 (12%) cases, endodontic treatment in 86 (8%) cases, and trauma in 74 (6%) cases. However, the arch was not mentioned. It was widely argued about whether gender or age gets more affected, and whether female or male is the predominant gender. Therefore, the results show that females (40-55.4 years) have a greater predominance in PTTN than males [21]. In one of the papers, it was stated that the pathology is more common in female patients in their mid-40s, affecting primarily the maxillary molar and premolar regions [22]. It also stated that the average age of the patients with continuous neuropathic orofacial pain was 54.5 years, with a clear female predominance (86.9%, n = 20). This was in contrast to the results mentioned in another study that indicated no gender predilection in PTTN with more prevalence in the mandibular arch [14]. Moreover, In the study by Oshima et al., RCT is seen to cause PTTN in the maxillary and posterior areas and 14 patients (87.5%) had maxillary pain and two (12.5%) had mandibular pain [12]. Ten (62.5%) of the 16 patients in the study said their discomfort started right after retreatment, while five (31.3%) said it started right after pulp extirpation. One patient made no explicit mention of how their discomfort first started. In a study by List et al., of 46 cases of PTTN, 56% of patients experienced upper jaw pain, as opposed to 45% experiencing lower jaw discomfort [23]. The upper jaw ratio was observed to be 8:2, with the majority in the molar area ratio (5:3). In the study by Klasser et al., 3.1% of patients had persistent discomfort that was not odontogenic [14]. Non-odontogenic pain was found in 22 patients in the maxillary region, while in 17 patients it was found in the mandible.

Regarding the time between the appearance of PTTN and root canal treatment, the level of non-odontogenic pain after six months or more after root canal treatment was reported to be 34% (95%CI 1.4-5.5) [24,25]. In cases where the extent of resorption defects is not identified, such as internal/external communication with the root canal and external surface of the root, suspicion of a perforation communicating with the external root surface, and root fracture where there may be a potential communication of the root canal with external root surface, it was determined that the pain caused by PTTN happened due to nerve injury after failed endodontic treatment, lower molar teeth with root apices close to the inferior dental canal and/or mental foramen, dens invaginatus, periapical lesions, and other diseases (cysts). This can be determined as a consequence of a failed treatment with an expensive cost that can keep the patient in chronic pain for a long period of time. In the study by Philpott et al., 20% of the sample size with predictive factors for PTTN, including after RCT, female gender, history of chronic pain, teeth responsive to pulp test, teeth associated with preoperative pain, tooth crack prior to treatment, and diameter of preoperative radiolucency, demonstrated persistent pain [13]. This was supported by another study [26]. "Temporal Summation" reveals that there are no appreciable differences between PTTN and healthy controlled patients in terms of temporal summation. Patients with PTTN who underwent "conditioned pain modulation" showed noticeably less effective CPM than the control group. Response to mechanical stimulation, the responses to 26 g intraoral stimuli were similar in the PTTN and control groups. In terms of pain intensity scores in response to the cold application, there were no statistically significant differences between the PTTN and control groups. However, the duration of cold pain after the removal of the cold stimulus, or the "time for the sensation to subside," was significantly longer in patients with PTTN. Several chronic pain conditions, including fibromyalgia, tension headache, temporomandibular disorders (TMDs), migraine, and irritable bowel syndrome, have been linked to factors that led to less effective CPM, raising the possibility that impaired CPM plays a part in the development and maintenance of chronic pain. In the retrospective study by Peñarrocha et al, the antecedents of trauma included extraction of an impacted lower third molar (34 patients, or 54%), followed by a straightforward extraction (nine patients), endodontic treatment (one patient) [16]. Pain was reported by four males and 32 females in their mid-40s, and age and arch were not mentioned.

In the retrospective study by De Poortere et al., the following were the statistically significant connections between various treatment subgroups and PTTN: the primary method of injury (n = 9, 31%) was implant surgery, which was followed by third molar extraction (n = 6), facial fractures (n = 4), bilateral sagittal split osteotomy (BSSO) (n = 4), and endodontic therapy (n = 3) [27]. Three to six percent of endodontic treatment patients have PTTN, with the prevalence of the condition being highest in women (40 patients), according to the results of two studies mentioned in literature [17,28]. Of patients who were treated by endodontics, the majority were females and in the mid-40s age range according to a two-year retrospective cohort study by Klazen et al. [9]. Females were recorded substantially more often than males (P > 0.05). In the study by Renton and Yilmaz, it was seen that iatrogenic inferior alveolar nerve injuries (IANI) were brought on by temporomandibular surgery (60%), LA (19%), implants (18%), and endodontics (8%) [29].

According to Nixdorf et al., pain that was present six months after RCT can be categorized into: non-odontogenic: eight patients (seven from referred TMD pain and one from PDAP disorder), mixed: two patients (one PDAP, one TMD), maxillary teeth compromised 53% of treated teeth, and 89% were posterior teeth [18]. Only women, in their 40s, were mentioned. In a study by Pigg et al., after endodontic treatment, there was a 3.4% frequency of chronic non-odontogenic pain [30]. In the case-control study by Jacobs et al., 5.7% dealt with phantom pain; the ratio of women to men was 9:1. Most complaints were made about the molar area ratio (8:2), which affects the upper jaw ratio (5:3) [31].

In a study by Melek et al., orofacial discomfort was reported by the majority of PTTN (66%) and trigeminal neuralgia (TN) patients (80%). Patients with TN experienced pain attacks more frequently (71%) than those with PPTTN (28%), but TN patients were more likely to experience numbness (12%). For both the intermittent and affective pain dimensions, there was greater pain intensity in TN. Patients' oral HRQoL was significantly, but similarly, impacted by PPTTN and TN. Patients with TN experienced a condition burden that was noticeably more severe than those with PPTTN, with clear distinctions in the mobility and self-care domains. There was a tendency for TN patients (54%) to report signs of depression more frequently than PPTTN patients (36%), but clinically significant anxiety was similarly common in both groups (34% to 39%). Both groups' oral and overall health status were closely correlated with anxiety and pain self-efficacy [32].

## Conclusions

This review highlights a high-to-moderate level of evidence regarding PTTN after endodontic treatment. It was concluded that the pain was reported after endodontic treatment mostly arising from the non-odontogenic neuropathic origin, which also investigated the relationship between PTTN and RCT. This results in evident symptoms such as persistent pain after the endodontic treatment caused by a variety of incidents, such as impaired endogenous analgesia, pronociceptive pain modulation, and failed endodontic treatment, with more female predominance in their mid-40s. The pain difference between pulpal origin and PTTN will give a large insight for practitioners to properly diagnose PTTN without wasting any more time by doing other procedures that can negatively affect the patient’s tolerance level.
